# Surgical management of malignancy-associated carotid sinus syndrome: a case report and literature review

**DOI:** 10.1093/jscr/rjag035

**Published:** 2026-02-06

**Authors:** Nebojša Oravec, Rajat C Sharma, Nicholas Wiebe, Caitlin T Yeo

**Affiliations:** Department of Surgery, Section of General Surgery, University of Calgary, 1403 29 St NW, North Tower, Calgary, Alberta T2N 2T9, Canada; Department of Internal Medicine, Section of Cardiology, University of Calgary, 2500 University Drive, Calgary Alberta T2N 1N4, Canada; Department of Pathology and Laboratory Medicine, University of Calgary, 2500 University Drive, Calgary, Alberta T2N 1N4, Canada; Department of Surgery, Section of General Surgery, University of Calgary, 1403 29 St NW, North Tower, Calgary, Alberta T2N 2T9, Canada

**Keywords:** vagus nerve, baroreceptor, carotid sinus, syncope, vasovagal

## Abstract

A 59-year-old male was admitted to hospital for workup of a high-grade atrioventricular block, where he had recurrent episodes of symptomatic bradycardia and hypotension. Imaging performed in anticipation of pacemaker insertion demonstrated a right-sided neck mass obliterating the jugular vein and compressing the carotid artery. He continued to have episodes of hemodynamic instability despite transvenous pacing. Decision was made to proceed with excisional biopsy and surgical debulking as a therapeutic attempt for suspected mass effect on the carotid sinus. Intraoperatively, there was gross vagus nerve invasion and no hemodynamic response to direct manipulation of the carotid sinus. The vagus nerve was transected to eliminate tumor-related parasympathetic neurotransmission. Pathology revealed a squamous cell carcinoma. The patient had radiographic and clinical improvement after treatment with chemoradiation. In a small number of similar cases, surgical resection of the tumor or intracranial section of the vagus nerve resulted in resolution of vasomotor symptoms, in conjunction with adjunctive treatments.

## Introduction

Malignancies of the lateral neck do not typically present with symptoms prior to the identification of a discrete mass. Rarely, lesions in proximity of the carotid sinus manifest as hemodynamic derangements by inducing hypersensitivity of the baroreceptor reflex through a phenomenon known as malignancy-associated carotid sinus syndrome [1, 2]. As opposed to idiopathic carotid sinus hypersensitivity, tumors in vicinity of the carotid bulb produce symptoms either through mass effect or direct invasion of parasympathetic nerve fibers. We present a case wherein surgical debulking and sacrifice of the right vagus nerve was performed in a patient with a right neck mass causing recurrent hemodynamic collapse.

## Case report

**Figure 1:**
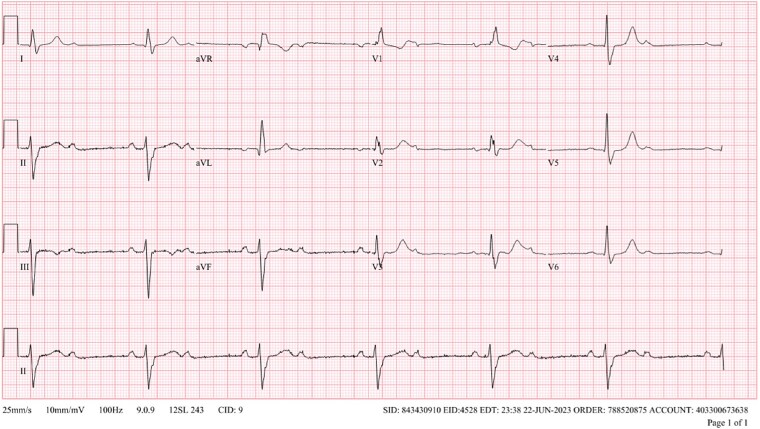
Initial ECG demonstrating bradycardia and second-degree bifascicular block and second-degree Mobitz type 2 AVB with 2:1 conduction.

A 59-year-old male with dyslipidemia and a 45 pack-year smoking history was urged to present to the emergency department by his primary care physician after routine electrocardiogram (ECG) demonstrated severe bradycardia (Fig. 1). He was admitted to the cardiology service and monitored with telemetry, where he was found to have a second-degree Mobitz type I atrioventricular block (AVB) with 2:1 conduction. During inpatient transthoracic echocardiogram, he became progressively bradycardic with ventricular rate of 30 beats per minute (BPM) and ECG demonstrating high grade AVB. He became hypotensive and eventually unresponsive. The patient regained consciousness after intravenous fluid resuscitation and was transferred to the cardiac care unit, where transvenous pacing was initiated via lead placement through the left internal jugular vein. His echocardiogram demonstrated moderate tricuspid regurgitation, normal left ventricular size and wall thickness, and mildly reduced systolic function (ejection fraction 40%–45%).

**Figure 2:**
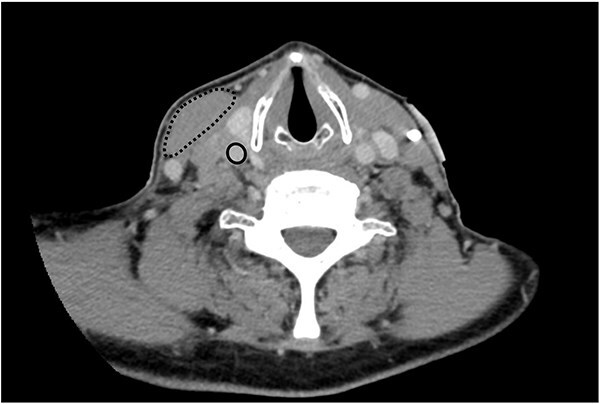
Axial plane image of contrast-enhanced CT scan demonstrating right-sided neck mass displacing the right sternocleidomastoid muscle (dashed line), occluding the right internal jugular vein (not seen) and compressing the right carotid artery (solid line) .

Computed tomography (CT) scan of his neck and chest was performed in anticipation of permanent pacemaker insertion, and demonstrated a mass at the level of the carotid artery measuring 3.6 cm in its maximal dimension causing occlusion of the right internal jugular vein and extrinsic compression on the right carotid artery (Fig. 2). During attempted ultrasound-guided lymph node biopsy, the patient developed transient hypotension, bradycardia, and altered consciousness requiring resuscitation. In the following days, the patient had recurrent episodes of symptomatic bradycardia and hypotension despite transvenous pacing, including an episode of pulseless electrical activity and bradycardic arrest requiring 3 minutes of cardiopulmonary resuscitation before return of spontaneous circulation.

**Figure 3:**
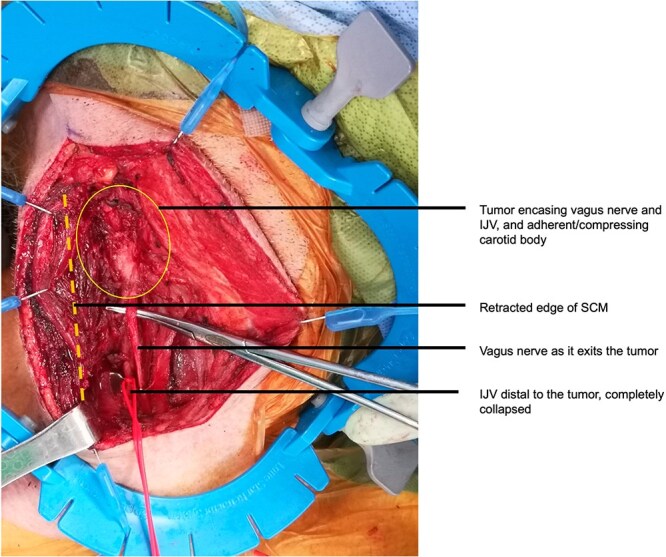
Intraoperative appearance of the tumor before debulking and resection of the right vagus nerve. IJV, internal jugular vein; SCM, sternocleidomastoid.

It was posed that mass effect from the tumor on the carotid sinus resulting in excess vagal tone could be responsible for the patient’s persistent bradycardia and syncope. Thus, surgical consultation was sought for consideration of excisional biopsy and tumor debulking. Intraoperatively, the sternocleidomastoid muscle was retracted laterally to reveal the right internal jugular vein, right vagus nerve, and right common carotid artery within the carotid sheath. All three structures were seen entering the main body of the tumor, which was firm and immobile at the carotid bulb. There was gross evidence of involvement of the carotid body (Fig. 3). The tumor was firmly adherent to the prevertebral fascia, the right external carotid artery, and the right internal jugular vein, without adequate visualization of the cranial ends of the vessels for vascular control. The carotid bulb was directly massaged in an attempt to elicit a cardiac response, but none was observed. It was hypothesized that the tumor was eliciting parasympathetic stimulation by direct invasion of vagus nerve fibers. After multidisciplinary intraoperative discussion including with the cardiac anesthesiologist, decision was made to transect the right vagus nerve to eliminate right-sided vagal parasympathetic neurotransmission to the heart. The risks associated with ipsilateral vocal cord paralysis (i.e. hoarseness, dysphagia, dyspnea, etc.) were weighed against the risk of persistent vasomotor symptoms and associated cardiac collapse. The nerve was transected distal to the carotid bulb within the carotid sheath, as it was not accessible proximally due to tumor bulk. An anterior portion of the tumor was excised from the remainder of the mass to partially debulk the carotid sinus and was sent for pathology.

**Figure 4:**
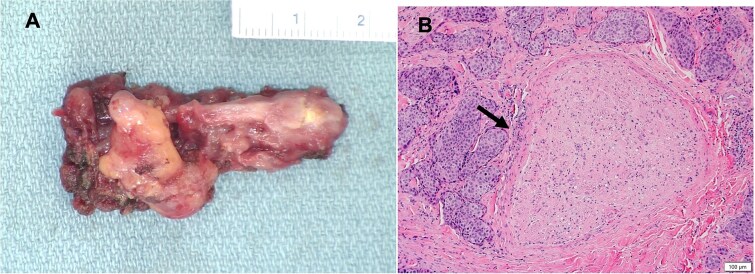
(A) Gross image of the excised portion of the vagus nerve; (B) Histopathologic image of hematoxylin and eosin-stained cross section of excised portion of vagus nerve showing squamous cell carcinoma (arrow) involving soft tissue adjacent to nerve.

Postoperatively, the patient continued to have episodes of profound bradycardia and hypotension, including an additional cardiac arrest, refractory to vasopressor infusions, and despite the presence of a well-functioning transvenous pacemaker with good ventricular capture at a rate of 90 bpm. He was started on fludrocortisone, midodrine, and caffeine. Full-length compression stockings and an abdominal binder were instituted to counteract vasoplegia. Eventually, he demonstrated some improvement and vasopressor infusions were weaned.

Histologic analysis of the right neck mass demonstrated a partially keratinizing squamous cell carcinoma with invasion into skeletal muscle and vasculature. The carcinoma involved soft tissue adjacent to the excised portion of the right vagus nerve (Fig. 4). Molecular testing was positive for high-risk human papillomavirus (HPV) subtype 16. Positron emission tomography scan demonstrated prominent activity at the base of the tongue and the tonsils, and extensive hypermetabolic cervical and mediastinal lymphadenopathy. Subsequent biopsy confirmed an HPV-associated squamous cell carcinoma originating from the right and midline base of the tongue. The patient’s disease was classified as locally advanced, stage II (T1N2M0). The case was discussed at multidisciplinary head and neck tumor board and recommendation was made for curative-intent radiotherapy (7000 cGy in 33 fractions) with 7 weeks of concurrent cisplatin chemotherapy.

Radiation treatment was initiated in hospital, and he was discharged 8 days later without further cardiac events. His total inpatient stay was 50 days, and he had no episodes of syncope after discharge. CT scan performed to assess treatment response after completion of chemoradiotherapy demonstrated decreased enhancement and volume of the infiltrative nodal conglomerate in the right neck, with no definitive residual tumor in the base of the tongue, and no new or progressive lymphadenopathy. Eight months after completion of chemoradiation therapy, he has discontinued all medications for blood pressure support. Continued cancer surveillance and cardiology follow up is planned.

**Table 1:** Cases of malignancy-associated carotid sinus syndrome treated with surgery

Author, year	Publication type	Age (years)	Sex	Vagal symptoms	Hemodynamic features	Oncologic diagnosis	Treatment of vagal symptoms	Surgery	Adjuvant therapy	Outcome
Muntz, 1983	Case series	61	M	Syncope	Hypotension, bradycardia	Metastatic epidermoid carcinoma of right epiglottis	Atropine, pacemaker	Subglottic laryngectomy with right radial neck dissection; intracranial section of glossopharyngeal and upper rootlets of vagus nerve	Adjuvant radiation	Complete resolution of syncopal episodes
		65	F	Syncope	Hypotension, bradycardia	Glomus vagale tumor	Atropine, lidocaine, dopamine, pacemaker	Distal vagus nerve section	None	Persistent hypotension responsive only to large doses of dopamine; tachyarrhythmia with frequent premature ventricular contractions
		71	M	Syncope	Hypotension, bradycardia	Epidermoid cancer of anterior commisure	Pacemaker	Total laryngectomy	Adjuvant radiation	Improvement in symptoms but ongoing bradycardia
		75	F	Syncope	Hypotension, bradycardia	Chondrosarcoma of larynx	None	Total laryngectomy and bilateral periarteriectomy	None	Complete resolution of symptoms after surgery
		78	M	Syncope	Hypotension, bradycardia	Epidermoid carcinoma of larynx	Dopamine, fludrocortisone, indomethacin, pacemaker	Total laryngectomy with neck dissection	Adjuvant radiation	Improvement in symptoms after radiation therapy
Frank, 1992	Case report	62	M	Fatigue, weakness, lightheadedness, unresponsiveness, syncope	Hypotension, normal heart rate	Metastatic squamous cell carcinoma of left piriform sinus	Fludrocortisone acetate 0.1 mg tid; abstinence from smoking, avoiding rightward head turning	Suboccipital craniotomy with sectioning of left glossopharyngeal nerve and upper rootlets of vagus nerve	Adjuvant chemotherapy and radiation	No further syncope for 6 months after surgery; death from respiratory failure
Janda, 2011	Case report	75	F	Syncope	None	Right carotid body tumor	None	Carotid body tumor resection	None	No symptom recurrence 15 months after surgery
Ye, 2022	Case report	35	F	Palpitations	Arrhythmia	Left cervical vagus schwannoma	None	Schwannoma resection with preservation of the vagus nerve	None	No symptom recurrence 1 year after surgery
Tzortzis, 2022	Case report	41	M	Syncope	Bradycardia	Right vagus nerve schwannoma	None	Schwannoma resection with preservation of the vagus nerve	None	No recurrence of symptoms after 12 months

## Discussion

The carotid sinus contains neurons which communicate with the vagal trunk, which in turn regulates heart rate and blood pressure via efferent vagal parasympathetic neurons [3]. Parasympathetic nerves synapse with ganglia located in the epicardium and atrial and ventricular septum, which project to cardiac muscle cells and the intrinsic cardiac conduction system [4]. The resulting effects are lowering of heart rate, slowing of AV conduction, reduction in ventricular contractility, and increase in the threshold for induction of ventricular fibrillation.

Carotid sinus syndrome is a phenomenon whereby hypersensitivity of the carotid baroreceptor reflex results in (i) a cardioinhibitory response with resulting bradycardia and asystole, (ii) a vasodepressor response causing reduction in systolic blood pressure by more than 50 mmHg, or (iii) a mixed response [5]. Carotid sinus syndrome due to a neck tumor was first described in 1933 [1]. Two theories are postulated for the mechanism of autonomic dysfunction: (i) extrinsic pressure by the tumor on the carotid body, and (ii) local invasion of the tumor into the carotid sinus, or vagus or glossopharyngeal nerves [6–8]. In our patient, although histopathologic analysis demonstrated no direct invasion of the transected portion of the vagus nerve, this portion was distal to the tumor. Given resection of the vagus did not alleviate cardiac symptoms, it was postulated that hypersensitivity of the carotid sinus reflex was maintained by diffuse tumor involvement of the right glossopharyngeal nerve and the carotid sinus, which was confirmed by gross inspection at the time of surgery.

A PubMed search related to vagal nerve sacrifice in patients with autonomic symptoms returned 68 articles (Supplementary File 1). Forward searching of reference lists of relevant articles revealed nine case reports or case series of malignancy-associated carotid sinus syndrome involving surgery as a treatment for the malignancy or associated vagal symptoms (Table 1) [9–13]. There were no relevant cohort studies or clinical practice guidelines. Seven out of nine patients with neck mass involving the carotid sinus and vasomotor symptoms underwent surgical tumor resection or debulking with preservation of the vagus nerve [6, 11–13]. One patient was treated with distal vagus nerve section alone [8], and another underwent tumor debulking with sectioning of left glossopharyngeal nerve and upper rootlets of vagus nerve [10]. All patients treated with surgery, whether involving vagus nerve transection or not, demonstrated resolution of autonomic dysfunction, although four of nine patients had adjuvant systemic or locoregional therapy.

Among patients who did not receive surgery, pacing alone or in combination with vasopressor infusion was successful in only 5 of 16 documented cases [6]. An important conclusion of this literature review is that idiopathic carotid sinus syndrome, for which pacemakers are more often effective, presents most often with pure cardioinhibitory response, whereas in the majority of malignancy-associated carotid sinus syndrome, a vasodepressor component is typically present [14–16]. In these cases, decreased venous return resulting from systemic vasodilation is the suggested mechanism for bradyarrythmias and asystole, as opposed to decreased firing of cardiac impulses transmitted to the sinoatrial node by increased vagal tone as would be the case for a cardioinhibitory syndrome. This may explain why pacing did not resolve our patient’s symptoms.

## Conclusion

Malignancy-associated carotid sinus syndrome is a rare clinical entity and there is a variety of described surgical and non-surgical treatments. Among a small number of published cases, all patients treated with surgery—with or without adjuvant systemic or locoregional therapy—had resolution of vagal symptoms. Pacing alone may not be effective in cases of malignancy-associated carotid sinus syndrome due to the vasodepressor response maintained by tumor invasion of the vagus or glossopharyngeal nerves.
